# United in stress, divided in response: grandparent–grandchild differences in COVID-19 protective attitudes and intergenerational conflict: findings from a Swiss survey

**DOI:** 10.3389/fsoc.2026.1847454

**Published:** 2026-07-17

**Authors:** Alexander Seifert, Benedikt Hassler

**Affiliations:** School of Social Work, Institute for Integration and Participation, University of Applied Sciences and Arts Northwestern Switzerland, Olten, Switzerland

**Keywords:** conflicts, family, generations, grandchildren, older adults, pandemic, protective measures, Switzerland

## Abstract

The COVID-19 pandemic has profoundly affected individual and family dynamics worldwide, with potential generational differences in experiences and attitudes. Understanding how these perspectives relate to intergenerational relationships and conflicts is essential for assessing long-term social impacts. This study examines retrospective evaluations of pandemic experiences and attitudes toward protective measures across family generations in Switzerland as well as post-pandemic developments in intergenerational relationships and conflicts. Data were collected between January and March 2025 from 1,205 respondents (M = 52.4 years, SD = 18.0; 55.7% female), each reporting on a randomly selected family member from another generation. Respondents were categorized as grandchildren (*n* = 274), children (*n* = 509), parents (*n* = 186), or grandparents (*n* = 236). Descriptive analyses revealed no significant generational differences in perceived pandemic stress. However, older generations—mostly grandparents—expressed stronger support for protective measures than younger generations—mostly grandchildren; this pattern remained significant in the multivariate analyses. Most respondents reported stable, positive intergenerational relationships. Notably, grandchildren reported improved relationships with grandparents more often than children did with their parents. Regression analyses further indicated that compared to grandchildren, grandparents were more likely to report that intergenerational conflicts had been less frequent during the pandemic than at the time of the survey. Overall, while stress perceptions were similar across generations, older generations—particularly grandparents—showed greater support for protective measures and reported fewer conflicts. Future research should examine how generational differences in risk perception shape long-term family cohesion beyond crisis contexts.

## 1 Introduction

The COVID-19 pandemic constituted an unprecedented global crisis that profoundly disrupted everyday life and social relations across all age groups (Lupton, 2022). Beyond its immediate health implications, the pandemic was widely experienced as a significant psychological and social stressor, affecting individuals regardless of age or generational belonging. Research has consistently shown that both younger and older populations reported heightened levels of stress, uncertainty, and disruption to daily routines during this period, although the sources and perceptions of these burdens often differed across age groups (König and Isengard, 2022; Sullivan et al., 2024). While much of the existing literature has focused on individual-level outcomes or broader societal challenges, less attention has been paid to how these stressors are negotiated within the microcosm of intergenerational family members (Feinberg et al., 2022; Stokes and Patterson, 2020).

Families represent a central context in which societal crises are experienced, interpreted, and managed. Intergenerational relationships within families, in particular, are characterized by a dynamic interplay of solidarity, conflict, and ambivalence (Bengtson and Roberts, 1991; Bengtson et al., 2002). These relationships involve ongoing processes of negotiation, mutual support, and sometimes tension between generations whose life circumstances, values, and risk perceptions may differ (Szydlik, 2016). The COVID-19 pandemic created a unique situation in which such differences became especially salient, as public health measures directly affected intergenerational interactions and responsibilities (Ellerich-Groppe et al., 2021).

During the pandemic, protective measures, such as social distancing and contact restrictions, were often framed as necessary to protect more older vulnerable populations. This framing positioned younger generations—particularly children and grandchildren—as potential carriers of risk, thereby assigning them a degree of responsibility for safeguarding vulnerable older family members (Sieverding and Wallis, 2024). At the same time, these measures imposed substantial limitations on the everyday lives of younger individuals, including restrictions on social activities, education, work, and leisure. Within families, this could give rise to tensions, as younger generations might perceive these sacrifices as disproportionately burdensome, while older generations might either welcome the protective intent or feel ambivalent about the resulting social isolation (Kuball et al., 2024). Although the specific circumstances differed across the life course, the pandemic disrupted important social roles and activities for all age groups, including education and peer interactions among younger people, work and caregiving responsibilities among adults, and opportunities for social participation and community engagement among older adults, potentially contributing to increased loneliness and social isolation.

Empirical evidence suggests that the pandemic significantly altered patterns of intergenerational contact. While physical interactions declined, some families compensated through increased nonphysical communication, such as phone or video calls (Arpino et al., 2021a, b; Vergauwen et al., 2022). However, the ability to maintain such contact was unevenly distributed. Older adults, in particular, often face barriers related to digital literacy and access, leading to risks of both social and digital exclusion (Seifert et al., 2021). Consequently, reduced face-to-face contact cannot always be fully offset by digital means, contributing to feelings of loneliness and disconnection (König and Isengard, 2022). These challenges were especially pronounced in grandparent–grandchild relationships, which often rely on both physical and emotional closeness (Döring et al., 2024; Fu et al., 2026; Hurme et al., 2010; Li et al., 2026). Nevertheless, this period also posed significant challenges for children and adolescents, as restrictions on face-to-face interactions with peers occurred during critical stages of social and emotional development. Such disruptions may have had lasting implications for their well-being and developmental trajectories (Feinberg et al., 2022).

At the same time, the pandemic highlighted the importance of communication processes within families. How family members discussed risks, protective measures, and responsibilities played a crucial role in shaping both behaviors and relational outcomes (Gong et al., 2023). Differences in attitudes toward COVID-19 measures—ranging from strong support to skepticism—could exacerbate existing generational divides, leading to conflicts over appropriate behaviors and levels of precaution. Such tensions reflect broader societal debates but are often intensified within the intimate context of family relationships, where expectations of solidarity and mutual care are particularly strong (Harwood, 2024; Höppner et al., 2022). These tensions between younger and older generations may also foster ageism, reinforcing negative stereotypes and biases toward the respective out-groups (Ayalon et al., 2021). In the context of COVID-19, differing perceptions of risk, responsibility, and compliance with protective measures could intensify such mutual age-based prejudices, thereby further straining intergenerational relationships (Ayalon et al., 2021; Drury et al., 2022).

Importantly, intergenerational dynamics during the pandemic cannot be understood solely in terms of conflict. Many families also demonstrated resilience and solidarity, with younger individuals engaging in prosocial behaviors to support older relatives (Sieverding and Wallis, 2024), and digital communication fostering new forms of connection (Döring et al., 2024; Hwang et al., 2024). Nevertheless, the coexistence of solidarity and tension underscores the ambivalent nature of intergenerational relationships, especially under conditions of crisis.

While numerous studies examined family relationships during the acute phases of the COVID-19 pandemic, less is known about how family members retrospectively evaluate these experiences several years later. Such retrospective assessments provide valuable insights into which tensions, conflicts, and forms of solidarity remain salient beyond the immediate crisis and continue to shape intergenerational relationships. Against this backdrop, the present study focuses on intergenerational relationships within families as a key site for understanding how the pandemic was experienced and negotiated across generations. By examining retrospective reports on pandemic-related stress, attitudes toward protective measures, and changes in intergenerational relationships and conflicts, this study aims to shed light on the extent to which generational differences manifest within family contexts. Based on prior research on intergenerational solidarity and conflict, as well as generational differences in pandemic experiences, the authors hypothesized that grandparents experienced the pandemic as less stressful and were more supportive of protective measures than grandchildren (Bengtson et al., 2002; König and Isengard, 2022; Sieverding and Wallis, 2024). Additionally, drawing on empirical work on intergenerational dynamics, it was expected that older generations reported fewer conflicts during the pandemic than younger generations (Drury et al., 2022; Naim et al., 2023). The study aimed to contribute to a more nuanced understanding of how societal crises are reflected in and shaped by everyday family interactions and how these dynamics persist beyond the immediate context of the pandemic.

## 2 Methods

### 2.1 Participants

The study is based on a dataset of people aged 18+ who were living permanently in Switzerland at the time of the survey. The data were collected using the computer-assisted web interviewing (CAWI) method. Paper-and-pencil interviewing (PAPI) was also available upon request. The questionnaire, which consisted of closed-ended questions, was made available in three official languages of Switzerland (German, French, and Italian). The addresses were provided by the Federal Statistical Office based on the Swiss Household Panel. The survey started in January 2025 by sending a postal letter containing basic information about the study and a link to the online survey. In mid-February 2025, a postal reminder was sent to nonrespondents. Due to the low response rate among older adults, specifically in rural regions, a second reminder was sent in March 2025 to people over the age of 80 and to respondents in certain cantons. The analysis included only respondents who had a living family member (grandchild, child, parent, or grandparent). A total of 1,205 questionnaires were included in the analysis.

The mean age of the sample was 52.4 years (SD = 18.0, age range: 19–102), with 55.7% of the respondents being female, 44.3% being male, and 86.4% of them having Swiss citizenship. In the second part of the questionnaire, the respondents were asked to think of a specific family member and to answer questions about their relationship with that specific family member. There were precise instructions in the survey for selecting this family member. First, the respondents were asked to consider relatives from the most distant generation (grandparents or grandchildren). If no relatives from this generation were available, the next closest generation (parents or children) should be selected. Respondents were instructed to select, within the chosen generation, the relative whose birthday would occur next, ensuring a randomized selection. This procedure was chosen to reduce selection bias that might occur if respondents were asked to select the family member to whom they felt closest or most distant. The respective family member was called “Person A” in the survey. According to the selected family member, the respondents (Person X) were categorized as grandchild [*n* = 274; age (M = 31.8; SD = 9.3; range: 19–56)], child [*n* = 509; age (M = 48.6; SD = 10.2; range: 20–73)], parent [*n* = 186; age (M = 69.0; SD = 11.9; range: 37–93)], or grandparent [*n* = 236; age (M = 71.6; SD = 10.3; range: 44–102)] for all analyses. Table 1 shows a sample description.

**Table 1:** Sociodemographic characteristics of the sample.

Variable	*n*	%	M	SD
Gender				
Female	668	55.7		
Male	532	44.3		
Age^a^	1,200		52.4	18.0
Income^b^				
Difficult	82	7.1		
Neither easy nor difficult	514	44.5		
Easy	558	48.4		
Swiss citizenship				
Yes	1,037	86.4		
No	163	13.6		
Education				
Compulsory school	97	8.2		
Secondary school	778	65.4		
Academic degree	314	26.4		
Living alone				
Yes	194	16.3		
No	993	83.7		
Health status (1 = very bad to 5 = very good)	1,201		4.1	0.7
UCLA loneliness scale (1 = low to 5 = high)	1,175		1.9	0.8
Generational group				
Grandchild reporting on a grandparent	274	22.7		
Child reporting on a parent	509	42.2		
Parent reporting on a child	186	15.4		
Grandparent reporting on a grandchild	236	19.6		

a Age range: 19–102.

b “How well are you able to make ends meet financially?”.

### 2.2 Measures

To facilitate retrospective evaluations of the pandemic period by respondents, the years 2020 to 2022 were specified as the reference timeframe. This period had the most substantial impact on everyday life and served as the basis for all retrospective assessments. Attitude toward protective measures (regarding the pandemic) was measured using a 7-point Likert scale ranging from 1 = *very negative attitude* to 7 = *very positive attitude* (M = 5.11; SD = 1.82; *n* = 1,185). Covariates included age in years, gender (0 = *female*, 1 = *male*), Swiss citizenship (0 = *no*; 1 = *yes*), education (1 = *compulsory school* to 3 = *academic degree*), income (1 = *difficult to get by financially* to 3 = *easy to get by financially*), living alone (0 = *no*, 1 = *yes*), UCLA loneliness scale adapted from Hughes et al. (2004) (1 = *low* to 5 = *high*), health status (0 = *very poor to average*, 1 = *good or very good*), and general pandemic experience (1 = *not at all stressful* to 5 = *very stressful*).

The change in the frequency of conflicts with Person A since the pandemic was measured using a 5-point Likert scale ranging from 1 = *more conflicts during the pandemic* to 5 = *fewer conflicts during the pandemic* (M = 3.17; SD = 0.73, *n* = 829). Covariates included those mentioned above as well as variables with a specific focus on the relationship with Person A, such as the evaluation of the relationship with Person A at the time of the survey (1 = *almost only unpleasant* to 5 = *almost only pleasant*), the question of whether the relationship has improved since the pandemic (0 = *no*, 1 = *yes*), and physical distance to Person A (1 = *in the same household* to 6 = *more than two hours away*). In addition, the attitude toward protective measures was included as a covariate.

### 2.3 Analytic strategy

First, descriptive statistics were conducted to examine whether the overall experience of the pandemic and attitudes toward protective measures differed between generations. Group differences were tested using ANOVA. Second, simple linear regressions were used to examine bivariate associations with attitude toward protective measures as the dependent variable, followed by multiple linear regressions to control for covariates. Third, the change in conflict frequency with Person A was examined using descriptive statistics and simple and multiple linear regression. Linear regression was used because the dependent variables had five and seven ordered response categories and were treated as approximately continuous to facilitate interpretation, an approach considered acceptable for Likert-type variables with several categories (Norman, 2010). Missing data were excluded listwise. Analyses were conducted using SPSS 29 software.

## 3 Results

### 3.1 Experiences of the pandemic and attitudes toward protective measures

The survey respondents were asked how they experienced the pandemic in general. Possible responses ranged from 1 = *not at all stressful* to 5 = *very stressful* on a 5-point Likert scale. The mean value in the full sample was 2.89 (SD = 1.12). The mean values of the different generational groups showed only small differences, with the lowest mean among grandchildren (M = 2.88, SD = 1.10) and the highest among grandparents (M = 2.91, SD = 1.20). ANOVA indicated no significant differences between the four generational groups [*F*(3, 1,1920) = 0.228, *p* = 0.877].

Regarding attitudes toward protective measures, the results showed a generally favorable stance among respondents in Switzerland. Responses were measured on a 7-point Likert scale ranging from 1 = *strongly against* to 7 = *strongly in favor*. The mean score in the full sample was 5.11 (SD = 1.82). ANOVA indicated that group differences were statistically significant [*F*(3, 1,181) = 11.87, *p* < 0.001]. Post-hoc comparisons using Bonferroni adjustment revealed that grandchildren (M = 4.82, SD = 1.75) and children (M = 4.92, SD = 1.87) showed significantly lower mean scores than parents (M = 5.43, SD = 1.78) and grandparents (M = 5.60, SD = 1.69). Hence, older generations were more in favor of the protective measures during the pandemic than younger generations.

Simple and multiple linear regressions were conducted to determine the bivariate and multivariate effects on the differences in attitudes toward pandemic protective measures. Table 2 shows the bivariate relationship between the covariates and the dependent variable. In the simple linear regression models, “grandparent” (ref. *grandchild*) had a significant positive effect on the attitude toward protective measures, which means that the grandparents were more in favor of the protective measures than the grandchildren. In addition, a higher age was positively associated with a more positive attitude as well as higher education and income. Perceiving the pandemic as stressful was associated with a more negative attitude toward protective measures.

**Table 2:** Linear regression analyses with attitude toward pandemic protective measures as the dependent variable.

Parameter	Scale	Simple regression model	Model (1) (*N* = 1,089)	Model (2) (*N* = 1,088)
		Beta	Beta	Beta
Child/parent	*Yes* (ref. *grandchild*)	−0.06	0.05	–
Grandparent	*Yes* (ref. *grandchild*)	0.22^***^	0.18^***^	–
Age		0.20^***^	–	0.22^***^
Gender	*Male* (ref. *female*)	−0.03	−0.02	−0.03
Swiss Citizenship	*Yes* (ref. *no*)	−0.03	−0.06	−0.07^*^
Education	1–3	0.14^***^	0.14^***^	0.13^***^
Income	1–3	0.15^***^	0.13^***^	0.11^***^
Living alone	*Yes* (ref. *no*)	0.01	−0.02	−0.03
Loneliness scale	1–5 (*high*)	−0.04	0.02	0.05
Health status	(*Very*) *good* (ref. *bad to average*)	−0.01	−0.01	0.01
Pandemic experience	1–5 (*very stressful*)	−0.11^***^	−0.10^**^	−0.10^**^
Model fit			*F*(10, 1,078) = 8.57; *p* < 0.001	*F*(9, 1,078) = 12.06; *p* < 0.001
			Adj. *R*^2^ = 0.065	Adj. *R*^2^ = 0.084

Dependent variable: attitude toward protective measures during the pandemic (1, very negative attitude, 7, very positive attitude); ^*^p < 0.05; ^**^p < 0.01; ^***^p < 0.001.

Table 2 also presents the results of the two multiple linear regression models. In Model (1), the generational groups are included as two dummy variables (child/parent; grandparent) with grandchild as the reference category. Model (1) shows that after controlling for covariates, the positive effect of grandparent remained significant and positive. Among the covariates, education and income had a positive impact, indicating that higher income and higher education were positively associated with a more favorable attitude toward the protective measures during the pandemic. Furthermore, respondents with more negative experiences of the pandemic were more opposed to the protective measures. In Model (2), chronological age was included as a covariate instead of the generational groups to test whether age is linearly associated with the dependent variable. The results in Model (2) are very similar to Model (1). Chronological age was positively associated with a favorable attitude toward protective measures. Although the variable “child/parent” had no effect in Model (1), there seems to be a linear effect of age on the dependent variable. In addition to the covariates in Model (1), Swiss citizenship was negatively associated with the attitude toward protective measures in Model (2), indicating that Swiss citizens had a more negative attitude compared to foreign residents.

### 3.2 Development of intergenerational relationships since the pandemic

In addition to the general experience of the pandemic, this study examines the development of intergenerational relationships since the pandemic. The respondents were asked about the quality of their relationship with Person A during the pandemic and at the time of the survey. The comparison of the two answers allows for determining whether the relationship has worsened, improved, or remained stable. For the latter category, it was examined whether the relationship was classified as good, ambivalent, or bad.

Table 3 presents the changes in relationship quality with Person A across generational groups. Overall, a statistically significant but small association was found between generation and relationship change (Cramer’s *V* = 0.11, *p* < 0.001). Across all groups, the most common outcome was that relationships remained “(very) good,” as reported by 56.4% of grandchildren, 55.7% of children, 61.0% of parents, and 59.6% of grandparents. Improvements in relationship quality were significantly more frequently reported by grandchildren (25.0%) than by children (15.8%). Conversely, constant ambivalent relationships were significantly more often reported by children (13.9%) than by grandchildren (5.9%) and grandparents (3.6%). Only some respondents reported having a constantly bad relationship with Person A or a relationship that had worsened since the pandemic. Statistical testing revealed no significant differences between generations for these two categories, indicating that these patterns were evenly distributed across generational groups.

**Table 3:** Changes in relationship quality with Person A by generation.

Relationship change	Grandchild		Child		Parent		Grandparent	
	*n*	%	*n*	%	*n*	%	*n*	%
Improved	51^a^	25.0	69^b^	15.8	26^a,b^	15.1	39^a,b^	23.5
Remained (very) good	115^a^	56.4	244^a^	55.7	105^a^	61.0	99^a^	59.6
Remained ambivalent	12^a^	5.9	61^b^	13.9	12^a,b^	7.0	6^a^	3.6
Remained (very) bad	8^a^	3.9	12^a^	2.7	4^a^	2.3	1^a^	0.6
Worsened	18^a^	8.8	52^a^	11.9	25^a^	14.5	21^a^	12.7

Cramer’s *V* = 0.11, *p* < 0.001. Superscript letters indicate *post-hoc* comparisons with Bonferroni correction (adj. *p* < 0.05); cells within the same row sharing the same letter did not differ significantly.

Table 4 shows the changes in the frequency of conflicts with Person A since the pandemic. In the first step, simple linear regressions were conducted to assess bivariate associations. Being a grandparent (reference category: grandchild) and a higher age were both associated with a lower frequency of conflict with Person A during the pandemic. Among the covariates, higher education and income were negatively associated with changes in conflict frequency, indicating that individuals with higher income and education had experienced more conflicts with Person A during the pandemic than at the time of the survey. A more positive attitude toward protective measures was associated with fewer conflicts during the pandemic. Respondents who reported that their relationship with Person A had improved since the pandemic also reported experiencing more conflicts during the pandemic than at the time of the survey (after the pandemic).

**Table 4:** Linear regression analyses with change in frequency of conflicts with Person A since the pandemic as the dependent variable.

Parameter	Scale	Simple regression model	Model (3) (*N* = 715)	Model (4) (*N* = 714)
		Beta	Beta	Beta
Child/parent	*Yes* (ref. *grandchild*)	0.03	0.06	–
Grandparent	*Yes* (ref. *grandchild*)	0.15^*^	0.13^**^	–
Age		0.08^*^	–	0.10^*^
Gender	*Male* (ref. *female*)	0.00	0.00	0.00
Swiss Citizenship	*Yes* (ref. *no*)	−0.05	−0.06	−0.07
Education	1–3	−0.07^*^	−0.04	−0.04
Income	1–3	−0.08^*^	−0.10^**^	−0.10^**^
Living alone	*Yes* (ref. *no*)	0.06	0.06	0.06
Loneliness scale UCLA	1–5	0.03	0.05	0.05
Health status	(*Very*) *good* (ref. *bad* to *average*)	−0.04	0.03	0.03
Pandemic experience	1–5 (*very stressful*)	−0.07	−0.08^*^	−0.08^*^
Attitude toward protective measures	1–7 (*positive attitude*)	0.11^**^	0.09^*^	0.08^*^
Quality of relationship with A	1–5 (*almost only pleasant*)	−0.01	0.03	0.04
Relationship with A has improved	Yes (ref. *no*)	−0.13^***^	−0.14^***^	−0.14^***^
Physical distance to A	1–6 (*more distant*)	0.00	0.00	−0.01
Model fit			*F*(14, 700) = 3.56; *p* < 0.001	*F*(13, 700) = 3.65; *p* < 0.001
			Adj. *R*^2^ = 0.048	Adj. *R*^2^ = 0.046

Dependent variable: chance of conflict frequency with Person A (1, more conflicts during the pandemic, 5, fewer conflicts during the pandemic); ^*^p < 0.05; ^**^p < 0.01; ^***^p < 0.001.

Table 4 also presents the results of the two multiple linear regression models. In Model (3), the generational groups were included as two dummy variables (child/parent; grandparent) with grandchild as the reference category. Model (3) shows that, even after controlling for several covariates, the coefficient of “grandparent” remains significant and positive. Compared to their grandchildren, grandparents reported fewer conflicts during the pandemic than during the time of the survey (after the pandemic). Education was no longer significantly associated with the dependent variable, likely due to the correlation between education and income, which remained significant in Model (3). A higher income, a more negative pandemic experience, and the perception that the relationship with Person A had improved were associated with a higher frequency of conflict during the pandemic than at the time of the survey. However, a more positive attitude toward protective measures was associated with fewer conflicts during the pandemic. In Model (4), chronological age was included as a covariate instead of the generational groups to test whether age is linearly associated with the dependent variable. The results showed that higher age was associated with the perception that there were fewer conflicts with Person A during the pandemic. The coefficients of the other covariates in Model (4) were very similar to those in Model (3).

## 4 Discussion

The present study set out to examine whether family generations differed in their retrospective evaluations of the COVID-19 pandemic, their attitudes toward protective measures and their experiences of intergenerational relationships and conflict. Three central findings emerged. First, contrary to initial expectations, the perceived stressfulness of the pandemic did not differ significantly across generations. Second, older generations, especially grandparents, expressed more positive attitudes toward protective measures than younger generations, particularly grandchildren, even after controlling for sociodemographic and psychosocial covariates. In addition, higher levels of income and education were associated with more favorable attitudes toward the protective measures but also with a higher frequency of reported intergenerational conflicts, suggesting that resources and social positioning may influence both compliance orientations and the likelihood of engaging in or perceiving family tensions. Third, most respondents reported stable, positive intergenerational relationships, but grandchildren more often described improvements in their relationships with grandparents than did children with parents. At the same time, grandparents were more likely than grandchildren to report that intergenerational conflicts had been less frequent during the pandemic than at the time of the survey. Taken together, the results suggest that the pandemic was experienced as a broadly stressful event across generations but that generations differed in how they evaluated protection and how they navigated family tensions.

The absence of significant generational differences in general pandemic stress is noteworthy. Much of the literature has emphasized age-specific burdens during COVID-19, such as social deprivation and loneliness among older adults or disruptions in education, leisure, and social life among younger groups (Feinberg et al., 2022; König and Isengard, 2022; Sullivan et al., 2024). Our findings indicate that these burdens may have differed more in content than in overall intensity. In this sense, the pandemic may be understood as a shared crisis that affected all generations, even if the sources of strain were not the same. This result thus nuances the assumption that older adults necessarily experienced the pandemic as less stressful than younger family members.

In contrast, the finding that grandparents were more supportive of protective measures than grandchildren is consistent with both public discourse and prior empirical work. During the pandemic, protective measures were often legitimized through appeals to intergenerational solidarity and responsibility, especially the idea that younger people should modify their behavior to protect older and more vulnerable populations (Ellerich-Groppe et al., 2021; Sieverding and Wallis, 2024). Older adults may therefore have evaluated these measures more favorably because they were more directly positioned as beneficiaries of protection. At the same time, younger generations often bore substantial social costs from restrictions, which may have contributed to more critical views. This interpretation also fits research showing that discussions about COVID-19 within families shaped preventive behavior and that family communication patterns mattered to how such measures were understood and negotiated (Gong et al., 2023).

The conflict findings further underscore that intergenerational family relations during the pandemic were marked by both solidarity and asymmetry. Compared to grandchildren, grandparents were more likely to report that intergenerational conflicts had been less frequent during the pandemic than at the time of the survey, and increasing age was associated with an increase in conflicts since the pandemic. This is broadly in line with work showing that intergenerational relationships combine solidarity, conflict, and ambivalence rather than reflect only one dimension (Bengtson and Roberts, 1991; Bengtson et al., 2002). One possible explanation is that older generations may have been more likely to avoid overt confrontation in family relations, whereas younger generations experienced restrictions more strongly as interference with autonomy and daily life (Visser et al., 2024). The positive association between favorable attitudes toward protective measures and fewer conflicts also suggests that disagreements over the legitimacy of pandemic rules likely served as a concrete source of intergenerational tension. In this way, family relationships mirrored broader societal conflicts over risk, responsibility, and acceptable behavior during COVID-19 (Harwood, 2024; Höppner et al., 2022).

At the same time, the results should not be read as evidence of widespread intergenerational rupture. Most respondents described their family relationships as stably positive, and grandchildren were especially likely to report improvements in their relationships with grandparents after the pandemic. This resonates with research showing that crises can also reinforce intergenerational solidarity and prosocial orientation (Sieverding and Wallis, 2024). This is also compatible with studies demonstrating that when face-to-face contact declines, families often compensate through increased phone and video communication (Arpino et al., 2021a; Vergauwen et al., 2022). Digital forms of contact appear to have been particularly important for maintaining intergenerational bonds, and several studies suggest that digitally connected intergenerational ties are associated with better relational and psychological outcomes (Döring et al., 2024; Fu et al., 2026; Hwang et al., 2024). However, such adaptation was not equally available to everyone, as older adults with fewer digital resources were at a greater risk of digital exclusion (Seifert et al., 2021).

Finally, these findings also speak to the possibility that pandemic-related tensions reinforce age-based stereotypes. Disruptions in contact and disagreements over responsibilities can intensify ageism in both directions, especially when generations are framed as competing interest groups (Ayalon et al., 2021). The family, therefore, provides an especially revealing setting in which to observe how societal tensions become personalized and negotiated in everyday life. Overall, the study suggests that the pandemic did not divide generations in terms of stress itself but rather in how protection, responsibility, and conflict were interpreted within family members of different generations. Future research should examine the potential enduring implications of these generational disparities on family cohesion, mutual age stereotypes, and intergenerational solidarity, extending beyond the pandemic era. In the future, it would be beneficial to investigate whether differences in attitudes toward protective measures and intergenerational conflict continue to shape family relationships and perceptions of other age groups over time. The findings also have implications for practice and policy. From a practical standpoint, interventions that promote intergenerational communication and mutual understanding may assist families in navigating divergent perceptions of risk and responsibility during societal crises. At the policy level, public health communication strategies should acknowledge the potentially different experiences and priorities of family generations while avoiding narratives that may inadvertently reinforce intergenerational divisions. The enhancement of opportunities for intergenerational dialogue has the potential to strengthen both family resilience and social cohesion during future public health emergencies and other societal challenges.

Several strengths should be acknowledged, including the large and age-diverse sample, the explicit focus on intergenerational family relationships, and the randomized selection of the focal family member, which helped reduce selection bias and capture a broader range of relationship experiences. However, several limitations must be noted. First, the study design was cross-sectional. Therefore, any causal influence of the factors on the dependent variable must be interpreted with caution. Second, the retrospective nature of the study should be considered when interpreting the findings (Hipp et al., 2020). While this approach was necessary to examine how family members currently reflect on and evaluate their experiences during the COVID-19 pandemic, retrospective assessments may be influenced by recall bias and by subsequent experiences that shape the interpretation of past events. Third, both dependent variables in the two tested models exhibited low variance, and the second dependent variable had a substantial number of missing values due to non-responses to this question. This might lead to bias in the data and analyses. Fourth, the adjusted *R*^2^ of the two models was below 0.1, indicating that important explanatory variables were not included in the model. Fifth, the findings should also be interpreted in light of the Swiss context. Consequently, the findings may not be fully generalizable to countries that experienced different policy responses, stricter restrictions, or more polarized public debates during the pandemic. Sixth, key constructs such as attitudes toward protective measures and intergenerational conflict were operationalized using single-item measures, which may not fully capture the relational complexity and ambivalence of family interactions during the pandemic. Seventh, an additional conceptual limitation concerns the distinction between chronological age and family-generational position. In this study, the categories of grandchild, child, parent, and grandparent refer to respondents’ positions within the family structure and do not necessarily correspond to clearly separated age groups. Future research should further disentangle the effects of chronological age, life stage, and family-generational role in shaping pandemic-related attitudes and intergenerational conflict.

## Data Availability

The raw data supporting the conclusions of this article will be made available by the authors, without undue reservation.
